# Correction: Ongoing, rational calibration of reward-driven perceptual biases

**DOI:** 10.7554/eLife.96151

**Published:** 2024-02-05

**Authors:** Yunshu Fan, Joshua I Gold, Long Ding

**Keywords:** Rhesus macaque

Fan Y, Gold JI, Ding L. 2018. Ongoing, rational calibration of reward-driven perceptual biases. *eLife* **7**:e36018. doi: 10.7554/eLife.36018.

Published 10 October 2018

We noticed a few minor errors after publication.

First, in the logistic functions we used to describe the relationship between probability of a rightward choice and the motion coherence, the biases and the motion sensitivity, we accidentally dropped the ‘1+’ in the denominators in equation (1) and (2) in the method section during the revision process of the manuscript. However, the correct form of the functions was used in the actual analysis, so no results or figures were impacted.

The correct form of equation (1) is given here:

$$P_{rightwardchoice}=\lambda +\left(1-2\lambda \right)\times \frac{1}{1+e^{-Sensitivity\left(Coh-Bias\right)}}$$

The originally published equation (1) is shown for reference:

$$P_{rightwardchoice}=\lambda +\left(1-2\lambda \right)\times \frac{1}{e^{-Sensitivity\left(Coh-Bias\right)}}$$

The correct form of equation (2) is given here:

$$P_{rightwardchoice}=\lambda +\left(1-2\lambda \right)\times \frac{1}{1+e^{-Sensitivity\left(Coh-\left(Bias+Bias_{seq}\right)\right)}}$$

The originally published equation (2) is shown for reference:

$$P_{rightwardchoice}=\lambda +\left(1-2\lambda \right)\times \frac{1}{e^{-Sensitivity\left(Coh-\left(Bias+Bias_{seq}\right)\right)}}$$

Second, the ‘LR-Left blocks’ and ‘LR-Right blocks’ labels were inadvertently switched in Figure 1B.

The corrected Figure 1 is shown here:

**Figure 1:**
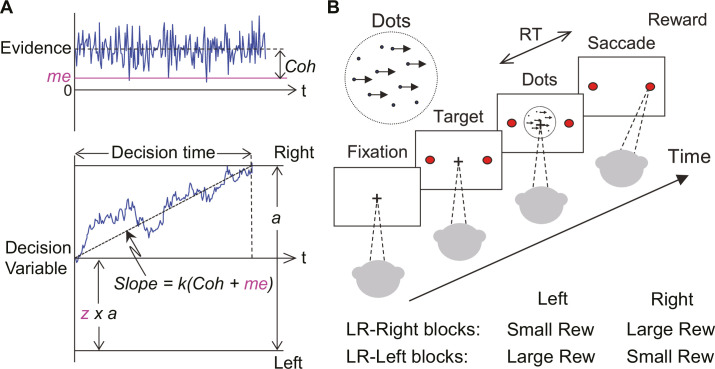


The originally published Figure 1 is shown for reference:

**Figure 2:**
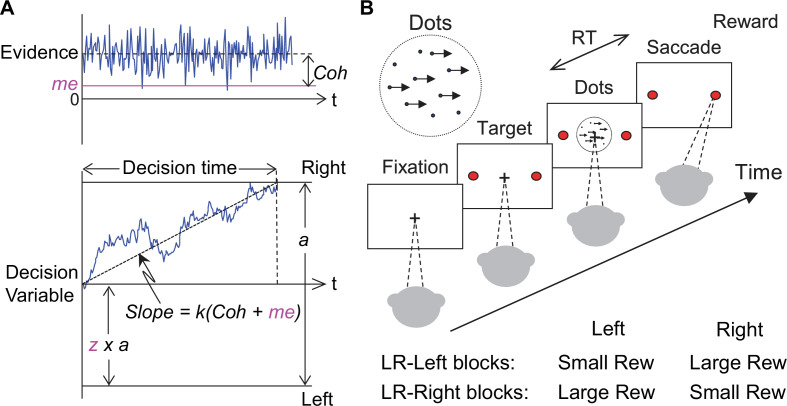


The article has been corrected accordingly.

